# Effect of Different Irrigating Solutions on Root Canal Dentin Microhardness—A Systematic Review with Meta-Analysis

**DOI:** 10.3390/jfb15050132

**Published:** 2024-05-16

**Authors:** Sunidhi Agarwal, Lora Mishra, Naomi Ranjan Singh, Rini Behera, Manoj Kumar, Ravishankar Nagaraja, Krzysztof Sokolowski, Barbara Lapinska

**Affiliations:** 1 Department of Conservative Dentistry & Endodontics, Institute of Dental Sciences, Siksha ‘O’ Anusandhan University, Bhubaneswar 751003, India; consendoids@soa.ac.in (S.A.); naomiranjansingh@soa.ac.in (N.R.S.); rinibehere@soa.ac.in (R.B.); 2 Department of Periodontics and Oral Implantology, Institute of Dental Sciences and SUM Hospital, Siksha ‘O’ Anusandhan University, Bhubaneswar 751003, India; manojkumar@soa.ac.in; 3 Department of Biostatistics, Vallabhbhai Patel Chest Institute, University of Delhi, Delhi 110021, India; rn@vpchest.du.ac.in; 4 Department of Conservative Dentistry, Medical University of Lodz, 251 Pomorska St., 92-213 Lodz, Poland; krzysztof.sokolowski@umed.lodz.pl; 5 Department of General Dentistry, Medical University of Lodz, 251 Pomorska St., 92-213 Lodz, Poland

**Keywords:** endodontic irrigants, root dentin microhardness, root canal irrigants, systematic review

## Abstract

The aim of this study was to evaluate the effect of different irrigating solutions as well as their combination and activation modes on root canal dentin microhardness. The protocol was registered in PROSPERO and PRISMA guidelines were followed. The structured question was as follows: “Which type of irrigating solution used in endodontic treatment causes more change in dentin microhardness?” The literature was screened via PubMed, Google Scholar, Scopus, and Science Direct. The last search was carried out in February 2023 with English language restriction. Two reviewers independently performed screening and evaluation of articles. A total of 470 articles were retrieved from all the databases, whereas only 114 articles were selected for full-text analysis. After applying eligibility criteria, 44 studies were evaluated and included in this review. The results showed that with increased contact time with irrigants, dentin microhardness decreases. Increased contact time with sodium hypochlorite (NaOCl) was associated with more reduction in dentin microhardness compared with other irrigants. Other irrigants, with the exception of distilled water, including EDTA, citric acid, herbal irrigants, glycolic acid, phytic acid, etc., in this study significantly decreased dentin microhardness. The maximum reduction in dentin microhardness was seen with 2.5% NaOCl after 15 min of contact time. The use of irrigating solutions alters the chemical composition of dentin, thereby decreasing its microhardness, which affects the clinical performance of endodontically treated teeth.

## 1. Introduction

Endodontic therapy relies crucially on the thorough chemo-mechanical preparation of the root canal system, which combines precise instrumentation with the application of effective irrigating solutions [1]. Irrigation is fundamental not only during the mechanical shaping but also subsequently, as it aids in removing microorganisms, tissue fragments, and dentinal debris via a flushing action [2]. It also helps avoid the accumulation of debris in the apical zone and the spread of infection to the periapical tissues [3].

The complex anatomy of root canals, with their varied shapes, narrow fins, isthmuses, and lateral extensions, often hinders complete debridement with instruments alone [4]. This underscores the importance of irrigation for ensuring the entire root canal is free from bacterial contamination and is an essential step for a successful endodontic outcome [5].

Commonly used endodontic irrigating agents include citric acid, hydrogen peroxide (H_2_O_2_), ethylenediaminetetraacetic acid (EDTA), sodium hypochlorite (NaOCl), and chlorhexidine (CHX) [6,7]. These substances offer a spectrum of beneficial actions, from antimicrobial effects to removing the smear layer and dissolving organic tissues [6]. Significantly, NaOCl, CHX, and EDTA are preferred due to their ability to dissolve organic tissue, eliminate the smear layer, and exhibit potent antimicrobial effects [8,9].

However, these solutions can also alter the chemical structure of dentin, particularly the calcium content in its hydroxyapatite crystals, which can subsequently influence key tooth properties like microhardness [10]. By evaluating dentin microhardness, we can infer changes in the physical and chemical properties of dentin such as the mineral content and modulus of elasticity of dentin [11]. Reduced dentin microhardness leads to a reduction in the modulus of elasticity of dentin [12].

Dentin microhardness measurement assesses the alteration in the calcium–phosphorus ration of the dentin structure. This provides indirect evidence of mineral loss or gain in the dental hard tissue [11].

This review rigorously investigates not only the direct effects of these solutions, but also delves into the methodologies, potential synergistic effects of combined irrigation protocols, and the role of activation methods. This comprehensive review of multiple databases aims to bridge the gap in the existing literature, providing a robust foundation for future research.

## 2. Materials and Methods

The protocol for this systematic review is registered with PROSPERO under the registration number CRD42022354739. This review was carried out following the Preferred Reporting Items for Systematic Reviews and Meta-Analyses (PRISMA) statement guidelines [13].

### 2.1. Eligibility Criteria

The eligibility criteria are presented in Table 1.

Focused PICO Question

The research question was formulated as follows:

- Population (P): extracted healthy human permanent teeth;
- Intervention (I): the application of various irrigating solutions in endodontic therapy;
- Comparison (C): various irrigating solutions;
- Outcome (O): dentin microhardness.

### 2.2. Literature Search

The search strategy followed PRISMA guidelines (Table 2). An electronic literature search was executed across four prominent databases: PubMed, Google Scholar, Science Direct, and Scopus, up to February 1, 2023. The search was restricted to articles published in the English language.

The search strategy incorporated the following keywords: “root canal dentin,” “radicular dentin”, “radicular dentinal surface”, “root dentin”, “irrigating solution”, “irrigation”, “root canal irrigation”, “root dentin irrigation”, “EDTA”, “CHX”, “chlorhexidine”, “NaOCl”, “sodium hypochlorite”, and “microhardness”. After identifying the relevant articles, a thorough screening process was undertaken to determine which studies would be included in the review.

Search results were imported into a reference manager software (Ravman, version 5, Boston, MA, USA), where duplicates were removed by S.A. and L.M. Titles and abstracts were then reviewed against the inclusion criteria, and studies meeting the criteria proceeded to full-text screening for qualitative synthesis.

### 2.3. Data Extraction

Data extraction involved three main categories: study characteristics, methodology, and outcomes/results. Study characteristics encompassed author names and publication years. Methodological variables included sample size, tooth specimens, tooth sectioning, irrigation protocol, microhardness test details (such as load and time), and percentage. Outcome variables comprised dentin microhardness levels at different points and changes in microhardness. Mean and standard deviation values were also documented from the included studies.

### 2.4. Quality Assessment

The quality assessment tool for in vitro studies (QUIN tool) checklist for reporting in vitro studies was used to evaluate the internal methodological quality (risk of bias) of the included studies resulting from the selection process. Each of the 12 parameters considered in the quality assessment tool was assessed for individual studies and then the percentage of complied items was calculated as (score ×100/2 × number of criteria applied).

## 3. Results

**Figure 1:**
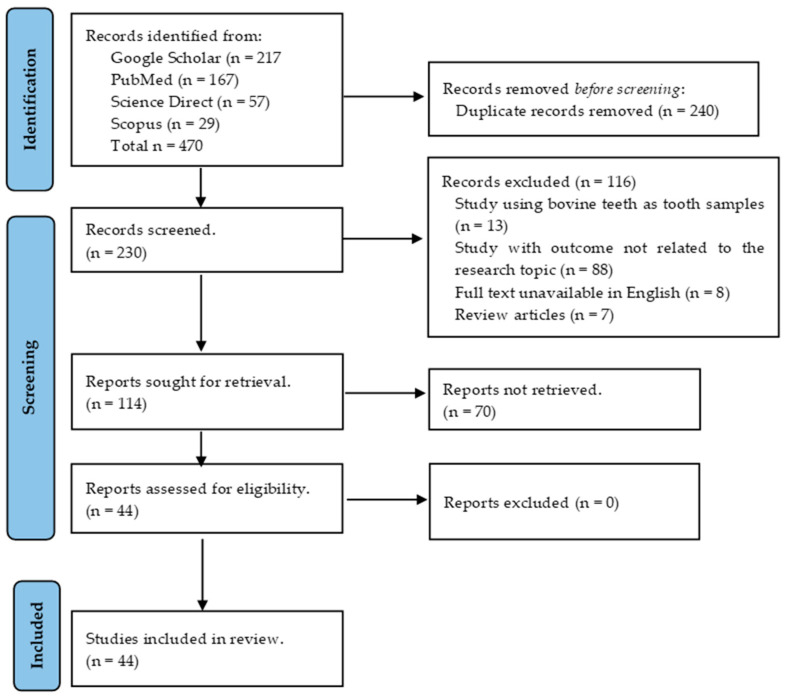
PRISMA flow diagram of literature search and selection process.

The initial electronic database search found 470 articles. After removing duplicates, the total was reduced to 230. Subsequent screening based on abstracts and titles resulted in a further assessment of 114 articles. Finally, 44 full-text articles met the eligibility criteria for this study (Figure 1).

### 3.1. Sample Size and Preparation

In total, 2267 healthy, extracted human teeth and 4534 sectioned tooth samples were utilized in the included studies. These predominantly employed single-rooted teeth like maxillary and mandibular incisors, canines, and mandibular premolars. The tooth sectioning process varied among studies using techniques such as diamond discs, low-speed and high-speed burs, cutting machines, and diamond saws. Most of the microhardness assessments were performed on longitudinally sectioned teeth with a few [11,14,15,16,17] opting for transverse cross-sections. Different storage media were used for tooth specimens including buffered saline [5,14,17,18,19,20,21,22,23,24,25] and 0.1% thymol [1,12,21,26,27,28,29] being the most common choices across different studies.

### 3.2. Microhardness Testing Tools

Microhardness testing was performed using either Vickers or Knoop diamond indenters. The majority of studies utilized Vickers diamond indenters, with a few exceptions that employed Knoop indenters [22,30,31,32].

### 3.3. Irrigating Solution Evaluation

Various irrigating solutions were evaluated for their impact on dentin microhardness (Table 3), with sodium hypochlorite and ethylenediaminetetraacetic acid being the most frequently studied solutions. They were tested at different concentrations and contact times.

### 3.4. Effect of Contact Time of Irrigating Solutions on Dentin Microhardness

The most significant reduction in microhardness was observed in the 2.5% NaOCl group with a 15 min contact time, with a Vickers Hardness Number value of 36.90 ± 2.46, compared to the control group that used distilled water, which had a microhardness value of 69.55 ± 4.65 VHN [21]. The least reduction in microhardness was seen in the 0.2% CHX group with a 15 min contact time, with a value of 61.58 ± 4.18 VHN, compared to the control group that used distilled water and had a microhardness value of 61.86 ± 11.70 VHN [20].

### 3.5. Effect of Various Irrigating Solutions on Dentin Microhardness

#### 3.5.1. Sodium Hypochlorite (NaOCl)

The reviewed studies used sodium hypochlorite concentrations ranging from 2.5% to 6%, with 2.5% NaOCl being the most tested [5,19,20,21,22,24,30,33,35,40,41,43]. At 15 min, 2.5% NaOCl significantly reduced dentin microhardness to 36.90 ± 2.46 VHN versus the control group’s 69.55 ± 4.65 VHN [21]. A 5% NaOCl solution showed the greatest reduction over 5 min, lowering microhardness to 45.69 ± 0.68 VHN from a pre-treatment level of 59.71 ± 2.31 VHN [41]. Concentrations of 3% and 6% NaOCl also decreased dentin microhardness to 43.59 ± 7.49 VHN and 64.3 ± 1.66 VHN, respectively, after 5 min [8,15]. Conversely, 1% NaOCl achieved only a slight reduction after 5 min but a notable decrease to 19.84 ± 12.11 VHN after 15 min, compared to the control saline’s 30.73 ± 10.60 VHN [28,32].

#### 3.5.2. Ethylenediaminetetraacetic Acid (EDTA)

Studies have assessed 17% EDTA as an irrigating solution, revealing it to be the second most examined. A notable decrease in dentin microhardness at 57.80 ± 4.83 VHN was observed using 17% EDTA for 15 min when compared to the control’s 69.55 ± 4.65 VHN [21]. A reduction was also seen with a 5 min exposure, while a 3 min application did not result in a significant change, yielding 39.28 ± 4.56 VHN versus the control’s 39.33 ± 3.18 VHN [48]. Lower concentrations of EDTA, specifically 5% and 15%, did not significantly alter microhardness after a 1 min contact time, with values recorded at 65.18 ± 5.52 VHN and 67.38 ± 3.35 VHN, respectively, against pre-treatment levels of 65.33 ± 6.88 VHN and 65.59 ± 6.65 VHN [33].

#### 3.5.3. Chlorhexidine (CHX)

The majority of studies focused on 2% chlorhexidine as an irrigant [8,16,28]. It reduced dentin microhardness the most to 20.89 ± 10.24 VHN after a 15 min contact time compared with the control (saline) group’s 30.73 ± 10.60 VHN [28]. A minimal reduction to 62.86 ± 1.57 VHN was noted after 5 min, versus the pre-treatment hardness of 65.09 ± 3.9 VHN [8]. Conversely, 0.2% CHX did not yield a significant change in microhardness, even after 15 min, when compared to the control group [20,21,36].

#### 3.5.4. Herbal Irrigants

Among the evaluated herbal irrigants, extracts of miswak stick, cashew leaves, and mango leaves showed no significant reduction in dentin microhardness when compared to the control group’s 0.30 ± 0.02 VHN [5]. Similarly, other herbal solutions like Triphala and MCJ did not significantly affect dentin microhardness after a 15 min contact time [1,12,16,36,39]. Triphala’s observed reduction was 43.60 ± 5.95 VHN, not markedly different from the control’s 55.07 ± 4.15 VHN, and it had a lesser impact than 5% NaOCl and 17% EDTA [1,16]. Combining MCJ with chlorhexidine did not show a significant reduction from the pre-treatment hardness, but some studies noted a reduction when MCJ was paired with EDTA [27,36,39]. Herbal irrigants such as 8% ethanolic Olea europaea extract and 2% ethanolic Morus nigra extract did lower microhardness significantly compared to their pre-treatment levels [40]. However, Sapindus mukorossi had no impact when compared to the control [37]. Interestingly, M. oleifera alone and combined with CHX resulted in an increased dentin microhardness compared to the control group [25].

#### 3.5.5. Citric Acid

A few studies have examined 10% citric acid as an irrigant, observing a reduction in dentin microhardness [11,14,46]. The greatest decrease was to 49.37 ± 3.89 VHN after 5 min, compared to the control group’s 62.6 ± 6.65 VHN [46]. It was found that there was no significant difference in the reduction of microhardness between 10% citric acid and 40% citric acid solutions [46]. In contrast, comparisons between 17% EDTA and 10% citric acid have yielded varied results. One study reported that 17% EDTA reduced microhardness significantly more to 34.7 ± 6.3 VHN than 10% citric acid at 41.8 ± 6.2 VHN after 5 min [14]. Another study found a minor reduction with 17% EDTA to 72.0 ± 1.3 VHN compared to 10% citric acid’s reduction to 48.3 ± 4.28 VHN after just 1 min [11].

#### 3.5.6. Peracetic Acid (PAA)

Peracetic acid demonstrated a reduction in dentin microhardness to 17.29 ± 3.71 KHN, which is comparable to the reduction observed with the NaOCl-EDTA-NaOCl sequence, at 17.95 ± 3.40 KHN [35].

#### 3.5.7. Other Irrigants

Several different irrigating solutions have been studied for their effects on dentin microhardness, including 0.2% chitosan, glycolic acid, Qmix, hydrogen peroxide, MTAD, CaOCl_2_, Chlor XTRA, Smear Clear, and Chloroquick, among others. (Table 3) For instance, 15 min of contact with 0.2% chitosan resulted in a reduction in microhardness to 44.65 ± 3.19 VHN from the initial 57.87 ± 1.60 VHN [12]. Also, a comparison between 17% EDTA and 0.2% chitosan showed that EDTA had a significantly larger effect, decreasing microhardness to 59.68 ± 0.30 VHN as opposed to 65.00 ± 0.49 VHN for chitosan [45].

Hydrogen peroxide demonstrated a decrease in microhardness after a 15 min contact time, with one study highlighting a substantial reduction to 57.20 ± 4.65 VHN compared to the distilled water control at 69.55 ± 4.65 VHN [21]. Studies comparing hydrogen peroxide and EDTA revealed no significant difference in their ability to reduce microhardness, although in one study, EDTA showed a greater effect than a combination of 3% H_2_O_2_/5% NaOCl [18,20,21].

Other irrigants like Chlor XTRA and a 5.5% sodium hypochlorite gel caused reductions similar to a 2.5% sodium hypochlorite solution [30]. Meanwhile, MTAD also decreased microhardness, notably to 45.78 ± 6.39 VHN after a 5 min contact time and was found to have a greater effect than the combination of NaOCl and EDTA [11,15,53]. However, 2% NaF did not present a significant difference compared with the control, while Smear Clear and QMix were similar in effectiveness to 17% EDTA [31,49]. Various concentrations of CaOCl_2_ showed reductions in microhardness, with 10% CaOCl_2_ marking the maximum decrease [41]. Glycolic acid, in its different concentrations, did not exhibit significant differences among the tested levels [48].

Moreover, the addition of surfactants to irrigating solutions was found not to alter root dentin microhardness [8,14,15,29]. EDTAC had a microhardness reduction value close to that of 17% EDTA, and the use of cetrimide with EDTA showed no significant difference from using EDTA alone [14,15,29].

Similarly, when using surface modifiers like Chlor-XTRA with NaOCl or REDTA (17% EDTA containing cetrimide), no significant differences were observed compared to the respective solutions without such additives [8]. Furthermore, nanoparticles such as CHX + CSNPs (chitosan-loaded nanoparticles) and MgO demonstrated a lower impact on reducing microhardness compared to a combination of 5% NaOCl with 17% EDTA [44].

#### 3.5.8. Activated Irrigating Solutions

Activation methods such as ultrasonic and laser agitation, including passive ultrasonic irrigation, were studied for their effects on dentin microhardness, yielding variable outcomes [23,26,32]. Irrigation solutions activated with ultrasonic agitation resulted in a decrease in microhardness to 23.6 ± 4.91 VHN, which was not significantly different from the group without agitation at 20.2 ± 3.36 VHN [23]. On the other hand, laser irradiation as an agitation method showed mixed results. One study indicated that laser activation led to less reduction in microhardness, at 50.6 ± 11.9 VHN, compared to the group not subjected to laser irradiation, which had a microhardness of 45.0 ± 9.7 VHN [32]. Yet, another study found that laser agitation used in conjunction with 17% EDTA and 5% NaOCl, followed by a rinse with distilled water, achieved the greatest reduction in microhardness at 18.62 ± 7.66 VHN when compared to a combination without laser agitation, which resulted in a microhardness of 20.2 ± 3.36 VHN [23].

### 3.6. Effect of Combination of Irrigants on Dentin Microhardness

Nineteen studies [11,17,19,22,23,26,27,29,31,32,34,35,36,38,43,44,47,52,53] have examined the synergistic effects of various combinations of irrigating solutions, with sixteen [1,11,17,19,22,31,32,33,34,35,38,43,44,47,52,53] of these specifically comparing the effects of NaOCl and EDTA in different concentrations. The combination of 2.5% NaOCl with 17% EDTA was most frequently analyzed [11,19,22,31,35,43,53] followed by 5% NaOCl combined with 17% EDTA [44,47,52]. The findings indicate that the mix of 2.5% NaOCl with 17% EDTA, which showed a microhardness reduction value of 30.7 ± 3.5 KHN, had a microhardness reduction comparable to the combination of 2.5% NaOCl with 10% citric acid, which resulted in a microhardness reduction of 31.5 ± 4.9 KHN after 30 min of contact time [22].

### 3.7. Quality Assessment

**Figure 2:**
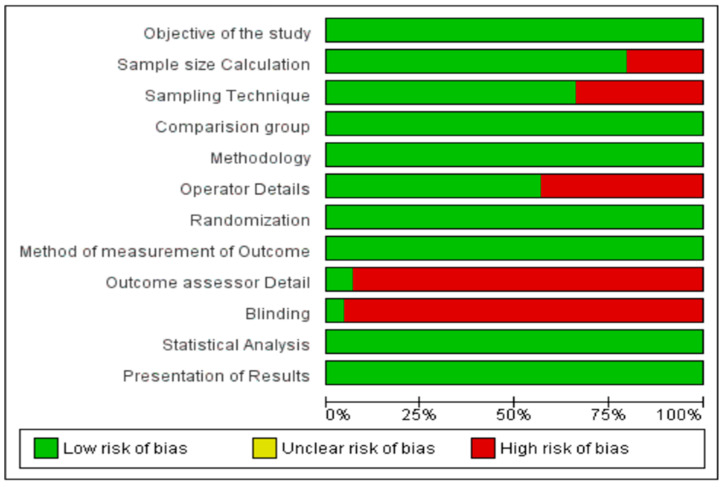
Quality assessment of included in vitro studies using the QUIN tool.

The quality of the in vitro studies was assessed using the Quality Assessment Tool For In Vitro Studies (QUIN tool) checklist. The checklist includes 12 items which covers elements like the clarity and appropriateness of the study’s objectives, the detailed characterization of the experimental model and conditions, the justification of sample sizes, the standardization of procedures, reproducibility of results, adequacy of statistical analysis, and transparency in reporting findings. The checklist aims to identify potential biases and determine the extent to which a study adheres to established scientific standards. Using such a tool in a systematic review ensures that conclusions are drawn from high-quality data, thereby contributing to the robustness of the evidence base in the field of study. The findings are summarized in Figure 2. The 44 studies assessed generally exhibited a consistent level of quality and a similar risk of bias. Most studies provided comprehensive rationales and clear objectives or hypotheses, and they typically detailed methodologies with defined study groups and outcome measures.

Method of measurement of outcome and the randomization process, presentation of results, and statistical analysis were uniformly reported. Only three studies discussed outcome assessor detail and two studies mentioned blinding. The percentage of checklist items met in the quality assessments ranged from 60% to 90% among the included articles.

### 3.8. Risk of Bias in Included Studies

**Figure 3:**
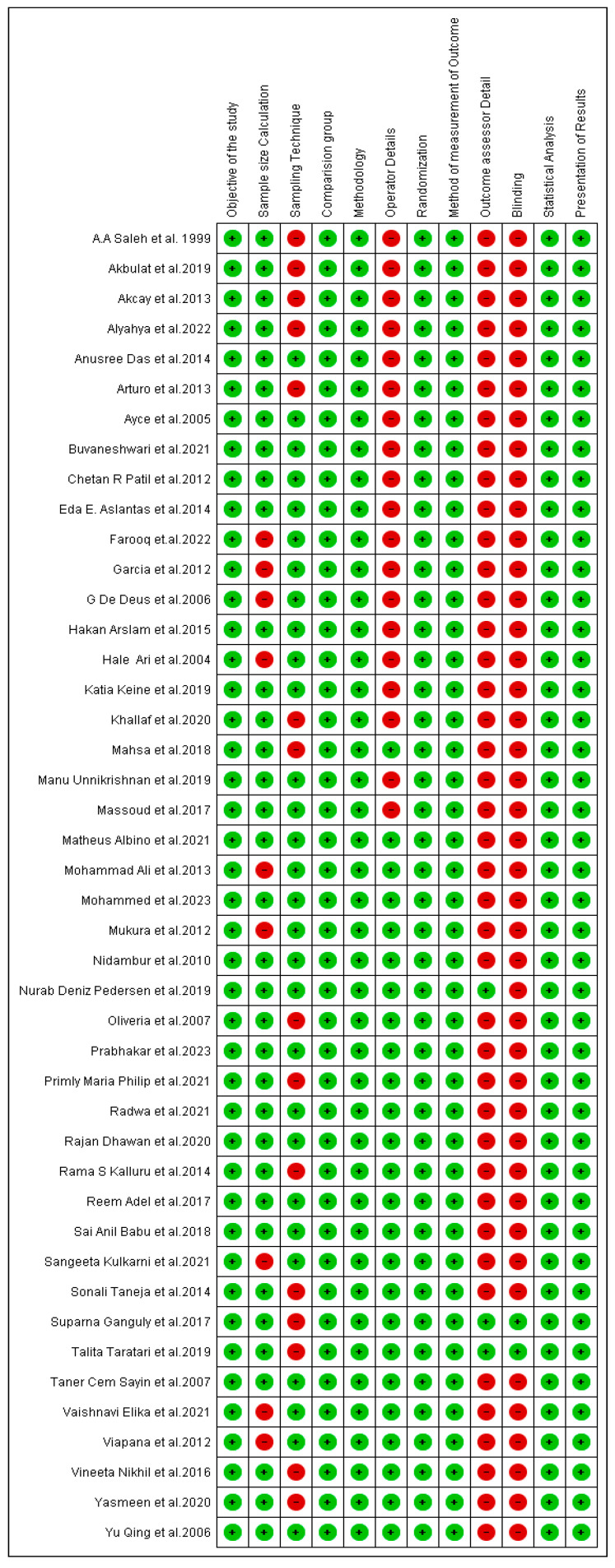
Risk of bias item for each included study using the QUIN tool.

The risk of bias assessment using the QUIN tool with 12 items categorized twelve studies as low risk, and the remainder as medium risk (Figure 3).

### 3.9. Meta-Analysis

**Figure 4:**
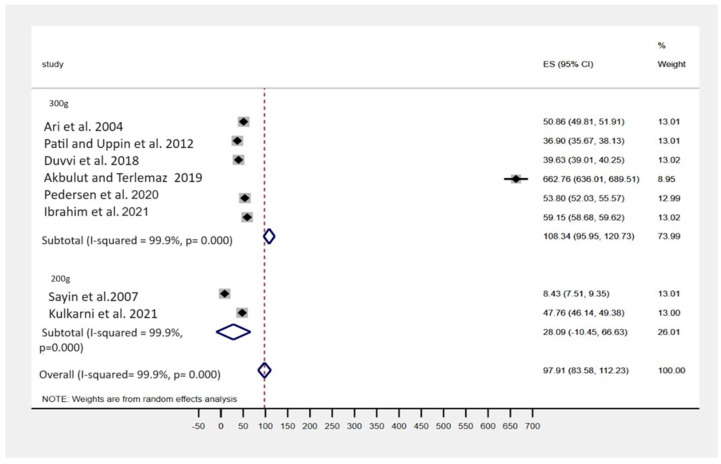
Forest plot showing the reduction in dentin microhardness using 2.5% NaOCl as an irrigating solution under 300 g [20,21,24,33,40,41] and 200 g of load [31,43].

The forest plot (Figure 4) shows the effect of 2.5% NaOCl on dentin microhardness under two different load conditions (300 g and 200 g). The studies indicate varying levels of reduction in dentin microhardness. Ari et al. [20] reported a mean reduction of approximately 50.86 ± 2.1 under 300 g of load. Patil and Uppin [21] reported a mean reduction of approximately 36.9 ± 2.46 under the same conditions. These results suggest that 2.5% NaOCl under a 300 g load has a significant impact on reducing dentin microhardness.

## 4. Discussion

In this systematic review and meta-analysis, we aimed to comprehensively assess the effect of various irrigating solutions on dentin microhardness, considering different contact times and concentrations. We synthesized data from 44 studies that met our eligibility criteria, examining a variety of irrigation solutions, including sodium hypochlorite (NaOCl), ethylenediaminetetraacetic acid (EDTA), chlorhexidine (CHX), herbal irrigants, citric acid, peracetic acid (PAA), and other novel irrigants. Our review also considered the activation methods used to enhance the effect of these solutions.

The inclusion of in vitro studies in this review helped in the detailed analysis of microhardness reduction by various irrigating solutions in a larger number of dentin samples than possible in human or animal trials.

This holds potential significance in the selection of endodontic irrigants as irrigants alter the chemical composition of dentin and can cause the formation and initiation of microcracks in dentin during endodontic procedures [53].

Success in the clinical performance of endodontically treated teeth is determined by the lesser impact of irrigating solutions on dentin microhardness as compared to the control group as a decrease in the microhardness of dentin weakens the tooth structure [34].

### 4.1. Study Quality and Risk of Bias

The quality assessment of the studies included in this systematic review was performed using the QUIN tool, a checklist of items for reporting in vitro studies specifically for dental related studies. Twelve studies [12,17,22,33,36,40,41,42,43,44,45,51] were categorized as having a low risk, with the rest positioned within the medium risk category [1,8,11,15,16,18,19,20,21,23,24,25,26,27,28,29,31,33,35,38,43,46,48,49,50,52,53] as detailed in Figure 3.

This variability in the quality of the studies is an important factor to bear in mind as it influences the interpretation of the results. The assessment showed that while some studies adhered closely to the quality criteria set by the checklist, others deviated to varying degrees. This finding underscores the necessity of a critical approach to data analysis since the risk of bias can impact the overall conclusions drawn from this systematic review.

### 4.2. Effect of Sample Preparation and Testing Method, Load, and Dwell Time on Microhardness of Dentin

Most of the studies opted for longitudinal sectioning of the tooth, which divides the root into buccal and lingual segments, exposing the superficial dentin (Table 3). This mirrors clinical scenarios and ensures direct contact of the irrigating solution with the superficial layer in the root canal lumen. This choice significantly impacts microhardness testing by providing a more accurate representation of clinical conditions during treatment, enhancing the reliability of the results [54].

The hardness test measures the resistance of dentin to deformation caused by the penetration of an indenting stylus. The microhardness test is easy, quick, and requires only a tiny area of specimen surface for testing. The mineral content of dentin contributes to its hardness. Any irrigating solution which alters the Ca/P level of dentin alters the hardness value directly [55].

Nine studies [5,19,22,25,26,30,44,45,50] evaluated the microhardness value of the coronal, middle, and apical third separately. Four studies [19,44,45,50] concluded that there was a difference in the microhardness levels of the coronal, middle and apical thirds. Reductions in microhardness values were greater in the coronal third than the apical third. The possible reason for this could be that the microhardness of dentin depends on the tubular density which varies from one area to another on the root dentin surface. The tubular density affects microhardness, as the tubular density at the coronal section increases dentin microhardness decreases. The other studies which did not show differences in microhardness levels in the coronal, middle, and apical sections may have used a contact time of more than 10 min. This could have resulted in the overall deterioration of the internal structure of dentin to a significant extent [55].

The Knoop and Vickers testing methods differ in the shape of the indenter. The Vickers indenter penetrates approximately twice as far into the specimen as the shallower Knoop indenter [56] and is a widely accepted method as only one type of indentation is used for all types of surface treatment [12]. The Vickers Hardness Number is based on the mean of two diagonals, providing more reliable results, whereas the Knoop test relies only on one diagonal [12]. Therefore, most studies have used the Vickers Microhardness Test, except a few studies [22,30,31,32] which used the Knoop indenter.

The load applied during the microhardness testing of root canal dentin also plays a crucial role in the accuracy of results. Studies typically used loads ranging from 25 g to 300 g (Table 3). Due to dentin’s elastic or viscoelastic nature, microhardness values at very low loads might be affected. Higher loads create larger impressions, aiding in indentation size measurement. This variation in microhardness with load is termed the Indentation Size Effect (ISE), which can be either normal, where microhardness decreases with increasing load, or reverse, where it increases. Comparing microhardness values obtained at different loads is not straightforward due to the various factors contributing to ISE, such as measurement accuracy, indenter geometry, and uncertainties in indentation area estimation, along with dentin’s physical properties like elastic recovery or elastic–plastic deformation after indenter removal [57].

Another inconsistency in the methodologies of the included studies is that the load applied was often more than the root dentin can take. It has been reported that healthy caries-free coronal dentin microhardness ranges from 52 to 64 KHN or 46 to 53 VHN. The root dentin has less mineral density compared to coronal dentin. Therefore, a higher load of more than 100 g may be impractical for a softer surface in the pre–post experiment because, after treatment, it produces a larger impression than the optical microscope can measure. The lowest loads, as small as 10 g for dentin, can create Vickers diagonals longer than 20 µm [57].

Additionally, the variation of loading times (10, 15, and 20 s) might have contributed to heterogeneity in the microhardness values. A study performed to investigate the effect of indentation load and time on the Knoop and Vickers microhardness tests for enamel and dentin concluded that an indentation time of 10 s is sufficient for a permanent indentation on the tooth surface to take place.

It is evident from the results that there is no standard condition for dentin microhardness testing across the included studies. The heterogeneity in the selection of testing conditions depended on the researchers’ decisions. The broad variation of hardness values can be produced by factors such as specimen preparation, diagonal length reading error, variation in chemical composition, age, and location in the tooth.

### 4.3. Effect of Individual Irrigating Solutions on Microhardness of Dentin

In our systematic review, we meticulously examined the impact of various irrigating solutions on dentin microhardness, a critical aspect influencing the success of root canal treatments. Our comprehensive analysis revealed nuanced effects of each solution, shedding light on their potential implications in clinical practice.

Sodium hypochlorite (NaOCl) emerged as a potent agent for dentin microhardness reduction, particularly at a concentration of 2.5% [5,19,20,21,30,35,41,53]. The dissolution of intertubular dentin following NaOCl treatment led to tubule enlargement and increased vulnerability to structural compromise [27]. Moreover, our findings underscored the dose-dependent nature of NaOCl’s effect, with higher concentrations and prolonged exposure exacerbating dentinal erosion and microhardness reduction [8,31].

Conversely, ethylenediaminetetraacetic Acid (EDTA) demonstrated significant dentin-softening capabilities attributed to its chelating action on calcium ions [43]. However, the extent of softening varied with EDTA concentration, necessitating cautious consideration in treatment planning [12,20,22]. Notably, concerns regarding EDTA’s potential to stimulate matrix metalloproteinase release raised questions regarding its long-term impact on dentin integrity [58].

Chlorhexidine (CHX) showcased dual-action properties, exhibiting both antimicrobial efficacy and dentin-softening capabilities [16]. While 2% CHX solutions altered dentin microhardness by disrupting the calcium–phosphate balance, lower concentrations released gradually over time facilitated canal shaping and sealing without compromising structural integrity [8,28].

Herbal irrigants, including Triphala and MCJ, offered intriguing alternatives to conventional solutions, albeit with milder dentin-softening effects [1,12,36]. Triphala’s bacteriostatic properties, attributed to its citric acid content, and MCJ’s organic acids demonstrated potential for application in specific clinical scenarios [1,16]. However, further research is warranted to validate their efficacy and safety profiles.

Citric acid, known for its chelating and smear layer removal properties, exhibited notable effects on dentin microhardness [11,14]. Its softening capabilities, dependent on pH rather than concentration, presented intriguing comparisons with EDTA, highlighting the need for nuanced evaluations in clinical settings [46].

Additionally, our review identified diverse effects of other irrigating solutions, such as MTAD [11,15,42,53], chitosan [12,45,50], CaOCl_2_ [28,41], and QMix [42,45,48], on dentin microhardness. While some solutions showed promising results, further investigation is essential to elucidate their mechanisms of action and clinical implications comprehensively.

### 4.4. Effect of Activation Methods of Irrigants

Studies [23,32] have looked into different activation methods for irrigating solutions, like laser irradiation and ultrasonic agitation. Ultrasonic agitation was found not to change dentin microhardness [23]. Lasers, however, with wavelengths between 810–980 nm, showed varying effects, largely depending on the irrigation solution used [23].

Some research has shown that laser agitation, especially when used with EDTA, can demineralize dentin, leading to a softer dentin structure. The laser works by vaporizing the dentin’s organic matrix, creating pores and voids, which ultimately reduces its microhardness [59].

### 4.5. Effect of Combinations of Irrigants

In endodontic treatments, irrigants are often used sequentially to enhance root canal cleaning [1]. The combination of sodium hypochlorite and EDTA is a common regimen [17]. This duo has been widely studied and is favored due to its synergistic effect on dentin microhardness [11,19]. The use of NaOCl followed by EDTA creates an alkaline environment which increases EDTA’s efficiency in chelating calcium ions, thus leading to greater dentin demineralization [60].

The mechanism involves EDTA’s chelation process, which targets the inorganic component, while NaOCl disrupts the organic matrix of dentin. Together, they reduce microhardness by softening the calcified tissues [60]. Moreover, the combination of NaOCl and EDTA was found to be comparable to the use of NaOCl with citric acid, since both EDTA and citric acid serve as chelating agents that demineralize dentin and facilitate the removal of calcium ions, altering the tooth’s structural properties [22].

### 4.6. Limitations of the Study

The ilimitations of the study encompass inconsistencies within the included studies and the necessity for additional studies to ascertain the practical significance of the observed effects.

Variability in factors such as the range of loads used for microhardness testing, differences in dentin properties across specimens, mode of delivery of irrigating solution, and variations in measurement techniques may introduce inconsistencies in the results.

Another possible limitation is the immersion treatment as the volume of the irrigant in a root canal clinically is small compared with the immersing root dentin in irrigating solutions. The experiments were also performed at room temperature and not body temperature.

This diversity in methodologies and experimental conditions of the included studies should be carefully considered when interpreting the collective findings of this review.

### 4.7. Recommendations for Future Studies

For future studies aiming to assess the microhardness of root canal dentin, the following recommendations are proposed:

1. Standardize Load Range: the load while preforming microhardness tests should gradually increase from 10–50 g;
2. Control Indentation Time: Standardize the duration of indentation to 10 s to prevent variations in results due to differences in the duration of load application. Consistency in indentation time helps ensure reproducibility of results;
3. Account for Dentin Properties: Take into account the inherent variability in dentin properties, such as its elastic or viscoelastic nature, which can influence microhardness measurements. Consider controlling for factors like dentin age, source (human or animal), and storage conditions to minimize variability;
4. Use Consistent Measurement Techniques: employ standardized measurement techniques for assessing microhardness, such as Vickers or Knoop hardness testing, to ensure uniformity across studies;
5. Address Indentation Size Effect (ISE): Recognize the potential impact of ISE on microhardness measurements and consider its implications in the interpretation of results. Investigate the presence of normal or reverse ISE and its effect on dentin microhardness under different experimental conditions;
6. Report Methodological Details: Provide detailed descriptions of the experimental procedures, including the type of indenter used, the range of loads applied, indentation time, and any adjustments made to account for dentin properties or ISE. Transparent reporting facilitates reproducibility and enhances the reliability of study findings;
7. Consider Microstructural Analysis: complement microhardness measurements with microstructural analysis, such as scanning electron microscopy (SEM) or atomic force microscopy (AFM), to gain insights into the structural changes accompanying variations in microhardness.

### 4.8. Clinical Implications

Irrigant solutions do alter the microhardness of root dentin which impacts the outcome of endodontic treatments. Despite their benefits like debris removal, disinfection, and smear layer removal, these solutions can also compromise dentin’s physical properties, including microhardness. Reduced microhardness aids instrumentation but can weaken the root structure. Microhardness assessment offers insight into mineral substance changes in dental hard tissues.

## 5. Conclusions

The impact of various irrigants on dentin microhardness is complex, determined by factors such as their concentration, duration of contact, and inherent chemical characteristics. The broad variation of hardness values in the included studies is due to factors such as specimen preparation, diagonal length reading error, variation in chemical composition, age, and location in the tooth.

From the present systematic review, one can conclude that NaOCl and EDTA concentration and contact time with both the organic and inorganic components of dentin plays a significant role in the reduction of microhardness. Chlorhexidine also alters the calcium to phosphate ratio and influences dentin’s structural integrity.

Interestingly, natural alternatives like Triphala present a gentler option with fewer adverse effects. The properties of other irrigants, such as glycolic acid, phytic acid, and chitosan, reflect their respective chemical compositions. Moreover, the choice of activation method can modify the outcomes of these irrigants, either enhancing or mitigating their effects on the microhardness of dentin.

However, more research is required to understand the complex interaction of irrigating solutions on the physical and mechanical properties of dentin using standardized methodologies.

## Figures and Tables

**Table 1:** Eligibility criteria.

Inclusion Criteria	Exclusion Criteria
In vitro studies investigating different irrigating solutions and their impact on dentin microhardness.	Review articles, letters to the editor, clinical studies, and case reports/case series.
Studies published in the English language.	Articles investigating changes in other parameters (surface roughness, erosion, flexural strength, etc.) and not including the microhardness of dentin.
Studies conducted on permanent healthy human tooth specimens.	Studies involving deciduous human teeth and bovine teeth.

**Table 2:** Search strategy.

Database	Search Strategy
PubMed	(((((((((root canal dentin) OR (radicular dentin)) OR (radicular dentinal surface)) AND (irrigating solution)) OR (irrigation)) OR (root canal irrigation)) OR (EDTA)) OR (CHX)) OR (sodium hypochlorite)) AND (microhardness)
Google Scholar	root canal dentin OR radicular dentin OR radicular dentinal surface AND irrigating solution OR irrigation OR root canal irrigants AND EDTA AND CHX AND NaOCl AND microhardness
Science Direct	root canal dentin AND irrigating solution AND EDTA AND CHX AND NaOCl AND microhardness
Scopus	root canal dentin AND irrigating solution AND microhardness

**Table 3:** Root dentin microhardness after contact with different irrigating solutions—data extraction from included studies.

No	Author	Sample Size, Type of Teeth, and Section Used in Each Group	Irrigants	Contact Time (Minutes)	Load (g) Given during Testing and Dwell Time	Microhardness Value (Mean ± SD)						MHN Test
						Cervical		Middle		Apical		
						Pre Rx	After Rx	Pre Rx	After Rx	Pre Rx	After Rx	
1.	Tartari et al. 2013 [22]	45 SRT LS	Saline	30	25 and 15 s	46.6 ± 6.3	46.0 ± 5.2	46.9 ± 5.1	45.1 ± 3.7	47.9 ± 6.8	43.7 ± 7.3	KHN
			5% NaOCl + 18% HEBP	30		43.7 ± 5.0	36.2 ± 5.4	45.5 ± 5.5	35.7 ± 4.1	46.1 ± 3.7	40.0 ± 5.7	
			2.5% NaOCl	30		44.7 ± 3.5	38.7 ± 3.8	44.9 ± 5.0	39.8 ± 2.9	45.2 ± 2.8	40.7 ± 5.0	
			2.5% NaOCl + 17% EDTA	30 + 3		47.5 ± 6.4	30.7 ± 3.5	47.3 ± 3.7	34.5 ± 5.4	47.2 ± 3.6	35.3 ± 4.0	
			2.5% NaOCl + 10% CA	30 + 3		43.7 ± 3.4	31.5 ± 4.9	45.2 ± 3.5	31.4 ± 7.4	45.4 ± 7.0	30.2 ± 5.4	
			2.5% NaOCl + 9% HEBP	30 + 5		45.9 ± 4.8	41.4 ± 4.9	47.7 ± 4.6	42.6 ± 3.0	46.4 ± 6.1	39.6 ± 5.8	
			2.5% NaOCL + 17% EDTA + 2.5% NaOCl	30 + 3 + 3		47.5 ± 6.4	30.2 ± 3.91	47.3 ± 3.7	34.4 ± 5.4	47.2 ± 3.6	35.7 ± 5.2	
			2.5% NaOCl + 10% CA + 2.5% NaOCl	30 + 3 + 3		43.7 ± 1.8	31.9 ± 6.8	45.6 ± 2.9	29.8 ± 6.4	45.1 ± 7.5	28.0 ± 3.6	
			2.5% NaOCl + 9% HEBP + 2.5% NaOCl	30 + 5 + 3		45.9 ± 4.8	39.1 ± 4.76	47.7 ± 4.6	41.8 ± 4.2	46.4 ± 6.1	39.4 ± 4.9	
						**Pre Rx**			**After Rx**			
2.	Pedersen et al. 2020 [33]	24 Molars LS	2.5% NaOCl + 5% EDTA	20 + 1	300 and 20 s	66.01 ± 5.66			56.69 ± 1.21			VHN
			2.5% NaOCl + 15% EDTA	20 + 1		66.15 ± 5.58			59.76 ± 3.42			
			2.5% NaOCl	2		66.01 ± 5.75			53.80 ± 3.54			
			5% EDTA	1		65.33 ± 6.88			65.18 ± 5.52			
			15% EDTA	1		65.59 ± 6.65			67.38 ± 3.35			
			Saline	20		65.72 ± 8.17			65.33 ± 8.46			
3.	Dineshkumar et al. 2012 [34]	40 Mand PM LS	1.3% NaOCl + 17% EDTA	20 + 1	300 and 20 s				51.63 ±0.86			
			1.3% NaOCl + MTAD	20 + 5					42.85 ±0.99			VHN
			1.3% NaOCl + HEBP	20 + 5					53.74 ±1.18			
			Distilled water	20					66.65 ±1.04			
4.	Keine et al. 2019 [35]	40 SRT LS	1% PAA	15	25 and 10 s				17.29 ± 3.71			KHN
			2.5% NaOCl	15					7.90 ± 1.94			
			2.5% NaOCl + 17% EDTA + 2.5% NaOCl	15 + 3 + 1					17.95 ± 3.40			
			0.9% saline (control group)	15					0.37 ± 0.24			
5.	Saha et al. 2017 [12]	80 PM LS	3% NaOCl	15	300 and 20 s	57.15 ± 1.75			55.15 ± 1.86			VHN
			17% EDTA			56.88 ± 1.38			43.12 ± 2.51			
			6% MCJ			57.92 ± 1.78			56.91 ± 2.11			
			0.2% chitosan			57.87 ± 1.60			44.65 ± 3.19			
6.	Ari et al. 2004 [20]	90 Mand Ant LS	5.25% NaOCl	15	300 and 20 s				51.74 ± 6.03			VHN
			2.5% NaOCl						50.86 ± 5.08			
			3% H_2_O_2_						53.57 ± 5.52			
			17% EDTA						53.66 ± 3.87			
			0.2% bCHX						61.58 ± 4.18			
			distilled water (control group)						61.86 ± 11.70			
7.	Elika et al. 2021 [1]	40 SRT LS	Saline	15	200 and 20 s	55.98 ± 3.94			55.07 ± 4.15			VHN
			5% NaOCl + 17% EDTA			54.03 ± 5.88			48.00 ± 5.32			
			Triphala			47.40 ± 5.53			43.60 ± 5.95			
			Chloroquick			43.46 ± 4.43			38.80 ± 4.90			
8.	Asghari et al. 2018 [16]	88 Mand PM Transverse	distilled water	15	200 and 15 s				45.27 ± 7.25			VHN
			Triphala						44.96 ± 7.15			
			2% CHX						41.62 ± 5.23			
			5.25% NaOCl						38.12 ± 6.71			
9.	Prabhakar et al. 2013 [36]	16 Mand PM LS	0.2% CHX	15	300 and 10 s	51.59 ± 8.98			53.15 ± 8.20			VHN
			6% MCJ			54.40 ± 8.42			57.38 ± 6.10			
			6% MCJ + 0.2% CHX			58.94 ± 8.80			59.14 ± 7.34			
			Saline			52.70 ± 8.15			55.68 ± 6.86			
10.	Farooq et al. 2022 [37]	90 SRT LS	Sapindus mukorossi	15	300 and 10 s				60.07 ± 0.49			VHN
			17% EDTA						56.62 ± 0.72			
			distilled water						60.45 ± 0.35			
11.	Patil and Uppin 2012 [21]	120 Incisors LS	2.5% NaOCl	15	300 and 20 s				36.90 ± 2.46			VHN
			3% H_2_O_2_						57.20 ± 4.65			
			17% EDTA						57.80 ± 4.83			
			0.2% CHX						65.05 ± 4.29			
			Distilled water						69.55 ± 4.65			
12.	Oliveira et al. 2007 [28]	30 PM LS	Saline	15	50 and 10 s				30.73 ± 10.60			VHN
			2% CHX						20.89 ± 10.24			
			1% NaOCl						19.84 ± 12.11			
13.	Garcia et al. 2013 [30]	24 Max Canines LS	2.5% NaOCl solution	15	25 and 10 s	**Cervical**		**Middle**		**Apical**		KHN
						0.58 ± 11.32				0.58 ± 11.32		
			ChlorXTRA			0.67 ± 22.57				0.67 ± 22.57		
			5.5% NaOCl gel			1.03 ± 12.10				1.03 ± 12.10		
14.	Yaseen et al. 2020 [38]	16 SRT LS	5.25% NaOCl + 13% GSE	15 + 15	300 and 20 s	17.48 ± 2.53						VHN
			5.25% NaOCl + 17% EDTA	15 + 15		34.75 ± 1.61						
15.	Philip et al. 2021 [5]	16 Max Canines LS	2.5% NaOCl	10	200 and 20 s	0.11 ± 0.02		0.10 ± 0.01		0.13 ± 0.02		VHN
			Miswak stick extract			0.28 ± 0.01		0.27 ± 0.01		0.14 ± 0.02		
			Cashew leaves extract			0.28 ± 0.02		0.28 ± 0.03		0.29 ± 0.01		
			Mango leaves extract			0.27 ± 0.01		0.28 ± 0.01		0.28 ± 0.02		
			Saline (control)			0.31 ± 0.02		0.30 ± 0.02		0.30 ± 0.01		
16.	Massoud et al. 2017 [19]	40 Mand PM LS	2.5% NaOCl	5	25 and 10 s	10.0 ± 21.15		8.92 ± 1.08		8.36 ± 1.16		VHN
			17% EDTA + 2.5% NaOCl	10		32.98 ± 6.06		30.37 ± 8.02		29.56 ± 8.01		
			2.5% NaOCl + 2% CHX	10		19.15 ± 3.09		17.68 ± 2.52		17.18 ± 2.35		
			2.5% NaOCl + distilled water + 2% CHX	15		15.16 ± 1.25		13.82 ± 1.10		13.23 ± 1.01		
17.	Saghiri et al. 2013 [39]	100 SRT LS	2.5% NaOCl	10	100 and 20 s	52 ± 2.0						VHN
			6% MCJ + 17% EDTA	10 + 1		54 ± 2.1						
			6% MCJ	10		53 ± 2.2						
			2.5% NaOCl + 17% EDTA	10 + 1		52 ± 2.2						
			1.3% NaOCl + MTAD	20 + 5		45 ± 2.2						
			2% CHX	5		4.1 ± 1.1						
			Saline (control group)	5		55.0 ± 1.1						
						**Pre Rx**			**After Rx**			
18.	Ibrahim et al. 2021 [40]	54 SRT LS	2.5% NaOCl10	10	300 and 20 s	83.56 ± 2.97			59.15 ± 1.76			VHN
			8% ethanolic extract of Olea europaea			85.52 ± 1.06			58.90 ± 1.25			
			2% ethanolic extract of Morus nigra			82.66 ± 1.23			60.82 ± 1.135			
19.	Kulkarni et al. 2021 [31]	24 Ant LS	17% EDTA + 2.5% NaOCl	2 + 10	200 and 20 s	50.32 ± 2.3			47.76 ± 4.05			VHN
			Saline	2					54.39 ± 3.59			
			2% NaF	2					47.05 ± 2.21			
			2% CHG	2					69.05 ± 2.46			
20.	Aslantas et al. 2014 [8]	25 Mand 3rd Molars LS	17% EDTA	5	300 and 20 s	66.01 ± 5.51			56.76 ± 8.05			VHN
			REDTA			59.76 ± 3.28			50.44 ± 4.23			
			6% NaOCl			68.47 ± 1.96			64.3 ± 1.66			
			6% NaOCl with surface modifiers			58.71 ± 3.71			56.66 ± 4.27			
			2% CHX			65.09 ± 3.9			62.86 ± 1.57			
			CHX-Plus			60.26 ± 1.91			60.04 ± 4.80			
21.	De-Dues et al. 2006 [14]	16 Max Canines Transverse	17% EDTA	5	50 and 15 s	47.6 ± 7.3			34.7 ± 6.3			VHN
			17% EDTAC			49.9 ± 9.0			36.6 ± 3.8			
			10% Citric acid			47.3 ± 7.0			41.8 ± 6.2			
22.	Kalluru et al. 2014 [15]	40 Mand PM Transverse	17% EDTA	5	50 and 15 s	55.5 ± 8.4			23.88 ± 4.59			VHN
			17% EDTAC			48.9 ± 7.5			24.11 ± 6.79			
			3% NaOCl			54.1 ± 7.2			43.59 ± 7.49			
			MTAD			51.3 ± 7.0			45.78 ± 6.39			
23.	Duvvi et al. 2018 [41]	75 Mand PM LS	Saline (control group)	5	300 and 20 s	56.95 ± 3.40			53.91 ± 2.56			VHN
			2.5% NaOCl			50.50 ± 2.54			39.63 ± 1.24			
			5% NaOCl			59.71 ± 2.31			45.69 ± 0.68			
			5% CaOCl_2_			57.06 ± 2.66			42.65 ± 1.45			
			10% CaOCl_2_			56.96 ± 1.84			39.03 ± 2.17			
24.	Das et al. 2014 [27]	40 Incisors LS	5% NaOCl + 17% EDTA + 2% CHX	5 + 5 + 5	200 and 20 s	64						VHN
			6% MCJ + 17% EDTA	5 + 5		68.3						
			5% NaOCl + QMix	5 + 5		69.9						
			Distilled water	5		74.9						
25.	Dhawan et al. 2019 [42]	120 PM LS	NaOCL-Extra	5	200 and 20 s	60 ± 0.02						VHN
			Pro-EDTA			55 ± 4.21						
			MTAD			59 ± 0.01						
			QMIx			63 ± 0.01						
			CHX-Ultra			66 ± 5.21						
26.	Sayin et al. 2007 [43]	30 SRT LS	2.5% NaOCl	5	200 and 20 s	8.43 ± 2.58						VHN
			17% EDTA			21.59 ± 4.47						
			17% EGTA			10.56 ± 3.34						
			1% tetracycline hydrochloride			8.53 ± 3.39						
			15% EDTAC			7.91 ± 1.34						
			distilled water			3.42 ± 1.91						
			17% EDTA + 2.5% NaOCl			27.54 ± 5.05						
			17% EGTA + 2.5% NaOCl			13.19 ± 5.08						
			15% EDTAC + 2.5% NaOCl			11.81 ± 4.45						
			1% tetracycline HCl + 2.5% NaOCl			11.06 ± 3.76						
						**Cervical**		**Middle**		**Apical**		
27.	Abdelrhman et al. 2023 [44]	16 Max Incisors LS	Nano MgO	5	200 and 20 s	7.89 + 0.74		8.88 + 2.24		7.69 + 2.28		VHN
			CHX loaded chitosan	5		13.74 + 5.29		13.38 + 2.39		13.28 + 2.31		
			5.2% NaOCl + 17% EDTA	3 + 2		19.47 + 2.67		21.93 + 0.49		19.47 + 2.67		
			Saline	5		0.56 + 0.40		0.69 + 0.40		0.43 + 0.26		
28.	Abdelgawad and Fayyad 2017 [45]	40 Max Incisors LS	2.25% NaOCl	Not mentioned clearly	50 and 10 s	70.92 ± 0.83		66.84 ± 1.22		76.86 ± 1.85		VHN
			17% EDTA			55.24 ± 0.45		59.68 ± 0.30		65.24 ± 0.577		
			Qmix			60.86 ± 0.15		63.02 ± 0.49		69.72 ± 1.188		
			0.2% Chitosan			63.80 ± 0.62		65.00 ± 0.49		73.88 ± 0.79		
29.	Khallaf et al. 2017 [25]	100 PM LS	Saline	Not mentioned clearly	200 and 15 s	63.73 ± 2.85		73.10 ± 12.74		60.57 ± 3.16		VHN
			M. oleifera			79.03 ± 9.92		71.30 ± 3.02		83.90 ± 5.01		
			M. oleifera and CHX			65.33 ± 5.10		87.33 ± 7.15		95.60 ± 7.61		
			CHX			89.23 ± 6.22		82.87 ± 12.97		99.17 ± 2.36		
			NaOCl			72.30 ± 2.15		76.77 ± 3.24		61.37 ± 2.95		
30.	Alyahya et al. 2022 [46]	45 SRT LS	distilled water	5	300 and 15 s	62.6 ± 6.65						VHN
			EDTA			54.92 ± 6.96						
			BioAKt			54.5 ± 5.95						
			40% citric acid			51.31 ± 6.097						
			10% citric acid			49.37 ± 3.89						
31.	Qing et al. 2006 [17]	43 SRT Transverse	5.25% NaOCl + 3% H_2_O_2_	5	50 and 15 s	50						VHN
			5.25% NaOCl + SAEW	5 + 1		47						
			5.25% NaOCl + distilled water	5 + 1		49						
			5.25% NaOCl + SAEW	5 + 3		44						
			5.25% NaOCl + 14.3% EDTA	5 + 1		44.5						
32.	Viapiana et al. 2012 [32]	72 Canines Transverse	distilled water	5	25 and 10 s	51.7 ± 10.9						KHN
			1% NaOCl			51.1 ± 11.6						
			1% NaOCL + 17% EDTA			54.4 ± 11.7						
			without irradiation			45.0 ± 9.7						
			Laser at 1.5 W/100 Hz			49.7 ± 11.2						
			Laser at 3 W/100 Hz			50.6 ± 11.9						
33.	Taneja et al. 2014 [47]	10 PM LS	5% NaOCl+ DW	5 + 5	300 and 15 s	77.39 ± 2.16						VHN
			5% NaOCl + 17% EDTA			69.70 ± 4.14						
			5% NaOCl + 2.25% PAA			62.98 ± 8.17						
			5% NaOCl + Qmix			70.68 ± 4.97						
34.	Souza et al. 2021 [48]	160 Incisors LS	distilled water	3	300 and 20 s	39.33 ± 3.18						VHN
			17% EDTA			39.28 ± 4.56						
			Qmix			38.07 ± 4.01						
			10% GA			35.62 ± 3.47						
			17% GA			35.91 ± 3.24						
			25% GA			35.98 ± 3.38						
35.	Aranda-Garcia et al. 2013 [49]	24 Max Canines LS	distilled water	3	25 and 10 s	0.00 ± 2.77						KHN
			17% EDTA	3		0.40 ± 28.37						
			BioPure MTAD	5		1.94 ± 25.72						
			SmearClear	1		2.53 ± 15.14						
			Qmix	2		1.10 ± 41.13						
						**Cervical**		**Middle**		**Apical**		VHN
36.	Nikhil et al. 2016 [50]	15 SRT LS	1% phytic acid	3	200 and 10 s	43.09 ± 7.40		43.59 ± 7.58		42.75 ± 6.87		
			17% EDTA			46.01 ± 5.93		44.32 ± 4.12		44.2 ± 3.69		
			0.2% Chitosan			49.41 ± 5.56		48.38 ± 5.16		48.14 ± 4.63		
37.	Ballal el al. 2010 [51]	45 Max CI LS	17% EDTA	1	200 and 20 s	55.64		50.17		41.15		VHN
			7% maleic acid			52.85		48.75		52.85		
			0.9% Saline			67.73		67.53		66.45		
38.	Akcay and Sen 2012 [29]	25 Canines LS	5% EDTA	1	50 and 10 s	7.30 ± 8.35						VHN
			5% EDTA + 0.25% cetrimide			8.78 ± 4.05						
			5% EDTA + 0.50% cetrimide			9.01 ± 4.14						
			0.25% cetrimide			4.59 ± 2.84						
			0.50% cetrimide			7.77 ± 3.83						
39.	Saleh and Ettman 1999 [18]	18 Max Incisors LS	3% H_2_O_2_/5% NaOCl	1	100 and 15 s	51.30 ± 0.02						KHN
			17% EDTA			47.30 ± 0.02						
40.	Unnikrishnan et al. 2019 [11]	60 SRT Transverse	17% EDTA + 2.5% NaOCl	1	300 and 15 s	55.80 ± 3.65						VHN
			17% EGTA			72.67 ± 5.65						
			MTAD			53.5 ± 2.78						
			10% citric acid			48.30 ± 4.28						
			17% EDTA			72.00 ± 1.30						
41.	Akbulut and Terlemez 2019 [24]	72 SRT LS	2.5% NaOCl	1	300 and 20 s	662.76 ± 115.8						VHN
			17% EDTA			541.41 ± 150.96						
			2% CHX			683.55 ± 152.13						
42.	Arul et al. 2021 [26]	60 Max Incisors LS	NI: 5% NaOCL + 17% EDTA + 5% NaOCl	1	100 and 10 s	1.68 ± 0.34		1.8 ± 0.324		2.4 ± 0.37		VHN
			PUI: 5% NaOCL + 17% EDTA + 5% NaOCl			2.90 ± 0.424		2.74 ± 0.64		2.4 ± 0.50		
			EndoVac: 5% NaOCL + 17% EDTA + 5% NaOCl			4.48 ± 0.841		5.14 ± 0.57		4.85 ± 0.43		
			Endovac + PUI: 5% NaOCL + 17% EDTA + 5% NaOCl			5.06 ± 0.680		5.15 ± 0.54		4.82 ± 0.60		
43.	Arslan et al. 2015 [23]	40 Max Ant LS	distilled water	2	50 and 15 s	4.30 ± 4.10						VHN
			17% EDTA + 5% NaOCl + DW			20.20 ± 3.36						
			17% EDTA + 60 s ultrasonic agitation + 5% NaOCl + DW			23.60 ± 4.91						
			17% EDTA + 10 s agitation with laser + 5% NaOCl + DW			18.62 ± 7.66						
			17% EDTA + 20 s agitation with laser + 5% NaOCl + DW			21.13 ± 5.24						
			17% EDTA + 30 s agitation with laser + 5% NaOCl + DW			23.19 ± 5.08						
			17% EDTA + 40 s agitation with laser + 5% NaOCl + DW			27.84 ± 25						
44.	Eldeniz et al. 2005 [52]	45 Mand Ant LS	17% EDTA+ 5.25% NaOCl	2.5 + 2.5	300 and 20 s	53.11 ± 7.40						VHN
			19% citric acid + 5.25% NaOCl			46.35 ± 5.77						
			distilled water			69.73 ± 7.89						

SRT = single root tooth; Max = maxillary; Mand = mandibular; Ant = anterior; PM = premolar; CI = central incisor; LS = longitudinal section; NaOCl = sodium hypochlorite; EDTA = ethylenediaminetetraacetic acid; EGTA = ethylene glycol tetraacetic acid; EDTAC = EDTA + Cetavlon; DW = distilled water; MCJ = Morinda Citrifolia Juice; PAA = peracetic acid; CHX = chlorhexidine; CaOCl_2_ = calcium hypochlorite; GA = glycolic acid; NaF = sodium fluoride; Chloroquick = 5% NaOCl + 18% etidronic acid; HEBP = (1-hydroxyethylidene-1,1-bisphosphonate); H_2_O_2_ = hydrogen peroxide; Q-Mix = 2% CHX + 17% EDTA + detergent; NI = needle irrigation; PUI = passive ultrasonic irrigation; REDTA = cetrimide + EDTA; MTAD = 3% doxycycline, 4.25% citric acid, and detergent (Tween 80); NaOCl Extra = 6% NaOCl and surface modifiers; CHX-Extra = 2% CHX + surface modifiers; BioAkt = 4.8% citric acid, 0.003% silver electrolytes, detergents, water; Smear clear = 17% EDTA + cetrimide, surfactant; BioPure MTAD = 3% tetracycline isomer (doxycycline), 4.25% citric acid, 0.5% detergent; ChlorXTRA = sodium hypochlorite and surface modifiers (Triton X-detergent).

## Data Availability

The data presented in this study are available on request from the corresponding author.

## References

[1] Elika V., Kunam D., Anumula L., Chinni S.K., Govula K. (2021). Comparative Evaluation of Chloroquick with Triphala, Sodium Hypochlorite, and Ethylenediaminetetraacetic Acid on the Microhardness of Root Canal Dentin: An in Vitro Study. J. Clin. Transl. Res..

[2] Ali A., Bhosale A., Pawar S., Kakti A., Bichpuriya A., Agwan M.A. (2022). Current Trends in Root Canal Irrigation. Cureus.

[3] Gomes B.P.F.A., Aveiro E., Kishen A. (2023). Irrigants and Irrigation Activation Systems in Endodontics. Braz. Dent. J..

[4] Kumar K., Teoh Y.-Y., Walsh L.J. (2023). Root Canal Cleaning in Roots with Complex Canals Using Agitated Irrigation Fluids. Aust. Endod. J..

[5] Philip P.M., Sindhu J., Poornima M., Naveen D.N., Nirupama D.N., Nainan M.T. (2021). Effects of Conventional and Herbal Irrigants on Microhardness and Flexural Strength of Root Canal Dentin: An in Vitro Study. J. Conserv. Dent. JCD.

[6] Kandaswamy D., Venkateshbabu N. (2010). Root Canal Irrigants. J. Conserv. Dent. JCD.

[7] Surya raghavendra S., Napte B. (2015). Endodontic Irrigants—A Review. J. Dent. Allied Sci..

[8] Aslantas E.E., Buzoglu H.D., Altundasar E., Serper A. (2014). Effect of EDTA, Sodium Hypochlorite, and Chlorhexidine Gluconate with or without Surface Modifiers on Dentin Microhardness. J. Endod..

[9] Rath P.P., Yiu C.K.Y., Matinlinna J.P., Kishen A., Neelakantan P. (2020). The Effect of Root Canal Irrigants on Dentin: A Focused Review. Restor. Dent. Endod..

[10] Sayin T.C., Cehreli Z.C., Deniz D., Akcay A., Tuncel B., Dagli F., Gozukara H., Kalayci S. (2009). Time-Dependent Decalcifying Effects of Endodontic Irrigants with Antibacterial Properties. J. Endod..

[11] Unnikrishnan M., Mathai V., Sadasiva K., Santakumari R.S.M., Girish S., Shailajakumari A.K. (2019). The Evaluation of Dentin Microhardness After Use of 17% EDTA, 17% EGTA, 10% Citric Acid, MTAD Used as Chelating Agents Combined With 2.5% Sodium Hypochlorite After Rotary Instrumentation: An In Vitro SEM Study. J. Pharm. Bioallied Sci..

[12] Saha S.G., Sharma V., Bharadwaj A., Shrivastava P., Saha M.K., Dubey S., Kala S., Gupta S. (2017). Effectiveness of Various Endodontic Irrigants on the Micro-Hardness of the Root Canal Dentin: An in Vitro Study. J. Clin. Diagn. Res. JCDR.

[13] Page M.J., McKenzie J.E., Bossuyt P.M., Boutron I., Hoffmann T.C., Mulrow C.D., Shamseer L., Tetzlaff J.M., Akl E.A., Brennan S.E. (2021). The PRISMA 2020 Statement: An Updated Guideline for Reporting Systematic Reviews. BMJ.

[14] De-Deus G., Paciornik S., Mauricio M.H.P. (2006). Evaluation of the Effect of EDTA, EDTAC and Citric Acid on the Microhardness of Root Dentine. Int. Endod. J..

[15] Kalluru R.S., Kumar N.D., Ahmed S., Sathish E.S., Jayaprakash T., Garlapati R., Sowmya B., Reddy K.N. (2014). Comparative Evaluation of the Effect of EDTA, EDTAC, NaOCl and MTAD on Microhardness of Human Dentin—An In-Vitro Study. J. Clin. Diagn. Res. JCDR.

[16] Asghari V. (2018). Evaluation of the Effects of Triphala on Dentin Micro-Hardness as Irrigation Solutions. J. Ayurveda Holist. Med..

[17] Qing Y., Akita Y., Kawano S., Kawazu S., Yoshida T., Sekine I. (2006). Cleaning Efficacy and Dentin Micro-Hardness after Root Canal Irrigation with a Strong Acid Electrolytic Water. J. Endod..

[18] Saleh A.A., Ettman W.M. (1999). Effect of Endodontic Irrigation Solutions on Microhardness of Root Canal Dentine. J. Dent..

[19] Massoud S.F., Moussa S.M., Hanafy S.A., El Backly R.M. (2017). Evaluation of the Microhardness of Root Canal Dentin after Different Irrigation Protocols (in Vitro Study). Alex. Dent. J..

[20] Ari H., Erdemir A., Belli S. (2004). Evaluation of the Effect of Endodontic Irrigation Solutions on the Microhardness and the Roughness of Root Canal Dentin. J. Endod..

[21] Patil C.R., Uppin V. (2011). Effect of Endodontic Irrigating Solutions on the Microhardness and Roughness of Root Canal Dentin: An in Vitro Study. Indian J. Dent. Res. Off. Publ. Indian Soc. Dent. Res..

[22] Tartari T., de Almeida Rodrigues Silva E Souza P., Vila Nova de Almeida B., Carrera Silva Júnior J.O., Facíola Pessoa O., Silva E Souza Junior M.H. (2013). A New Weak Chelator in Endodontics: Effects of Different Irrigation Regimens with Etidronate on Root Dentin Microhardness. Int. J. Dent..

[23] Arslan H., Yeter K.Y., Karatas E., Yilmaz C.B., Ayranci L.B., Ozsu D. (2015). Effect of Agitation of EDTA with 808-Nm Diode Laser on Dentin Microhardness. Lasers Med. Sci..

[24] Akbulut M.B., Terlemez A. (2019). Does the Photon-Induced Photoacoustic Streaming Activation of Irrigation Solutions Alter the Dentin Microhardness?. Photobiomodul. Photomed. Laser Surg..

[25] Khallaf M.E. (2017). Effect of Two Contemporary Root Canal Sealers on Root Canal Dentin Microhardness. J. Clin. Exp. Dent..

[26] Arul B., Suresh N., Sivarajan R., Natanasabapathy V. (2021). Influence of Volume of Endodontic Irrigants Used in Different Irrigation Techniques on Root Canal Dentin Microhardness. Indian J. Dent. Res. Off. Publ. Indian Soc. Dent. Res..

[27] Das A., Kottoor J., Mathew J., Kumar S., George S. (2014). Dentine Microhardness Changes Following Conventional and Alternate Irrigation Regimens: An in Vitro Study. J. Conserv. Dent. JCD.

[28] Oliveira L.D., Carvalho C.A.T., Nunes W., Valera M.C., Camargo C.H.R., Jorge A.O.C. (2007). Effects of Chlorhexidine and Sodium Hypochlorite on the Microhardness of Root Canal Dentin. Oral Surg. Oral Med. Oral Pathol. Oral Radiol. Endod..

[29] Akcay I., Sen B.H. (2012). The Effect of Surfactant Addition to EDTA on Microhardness of Root Dentin. J. Endod..

[30] Garcia A.J.A., Kuga M.C., Palma-Dibb R.G., Só M.V.R., Matsumoto M.A., Faria G., Keine K.C. (2013). Effect of Sodium Hypochlorite under Several Formulations on Root Canal Dentin Microhardness. J. Investig. Clin. Dent..

[31] Kulkarni S., Mustafa M., Ghatole K., AlQahtani A.R., Asiri F.Y.I., Alghomlas Z.I., Alothman T.A., Alhajri F.F. (2021). Evaluation of 2% Chlorhexidine and 2% Sodium Fluoride as Endodontic Irrigating Solutions on Root Dentine Microhardness: An In Vitro Study. Eur. J. Dent..

[32] Viapiana R., Sousa-Neto M.D., Souza-Gabriel A.E., Alfredo E., Silva-Sousa Y.T.C. (2012). Microhardness of Radicular Dentin Treated with 980-Nm Diode Laser and Different Irrigant Solutions. Photomed. Laser Surg..

[33] Pedersen N.D., Uzunoglu-Özyürek E., Dogan Buzoglu H. (2020). Influence of Different Irrigation Protocols on Microhardness and Flexural Strength Values of Young and Aged Crown Dentin. Gerodontology.

[34] Dineshkumar M.K., Vinothkumar T.S., Arathi G., Shanthisree P., Kandaswamy D. (2012). Effect of Ethylene Diamine Tetra-Acetic Acid, MTAD^TM^, and HEBP as a Final Rinse on the Microhardness of Root Dentin. J. Conserv. Dent. JCD.

[35] Keine K.C., Kuga M.C., Coaguila-Llerena H., Palma-Dibb R.G., Faria G. (2020). Peracetic Acid as a Single Endodontic Irrigant: Effects on Microhardness, Roughness and Erosion of Root Canal Dentin. Microsc. Res. Tech..

[36] Prabhakar A.R., Basavraj P., Basappa N. (2013). Comparative Evaluation of Morinda Citrifolia with Chlorhexidine as Antimicrobial Endodontic Irrigants and Their Effect on Micro-Hardness of Root Canal Dentin: An: In Vitro: Study. Int. J. Oral Health Sci..

[37] Farooq A., Rahman Qazi F.U., Siddiqui J., Faraz S.A., Rasheed A. (2022). Comparison of an Experimental Root Canal Irrigant (Sapindus Mukorossi) and Ethylenediaminetetraacetic Acid on Microhardness of Human Dentin. J. Ayub Med. Coll. Abbottabad JAMC.

[38] Yassen G.H., Al-Angari S.S., Platt J.A. (2014). The Use of Traditional and Novel Techniques to Determine the Hardness and Indentation Properties of Immature Radicular Dentin Treated with Antibiotic Medicaments Followed by Ethylenediaminetetraacetic Acid. Eur. J. Dent..

[39] Saghiri M.A., García-Godoy F., Asgar K., Lotfi M. (2013). The Effect of Morinda Citrifolia Juice as an Endodontic Irrigant on Smear Layer and Microhardness of Root Canal Dentin. Oral Sci. Int..

[40] Ibrahim R.O., Salama R.A., Amin A.M. (2021). Can Ethanolic Leaf Extract of Olive or Black Mulberry Substitute Sodium Hypochlorite as a Root Canal Irrigant? An In Vitro Study. J. Contemp. Dent. Pract..

[41] Duvvi S.A.B., Adarsha M.S., Usha H.L., Ashwini P., Murthy C.S., Shivekshith A.K. (2018). Acomparative Assessment of Different Concentrations of Sodium Hypochlorite and Calcium Hypochlorite on Microhardness Of Root Canal Dentin—An In Vitro Study. Int. J. Oral Care.

[42] Dhawan R., Gupta A., Dhillon J.S., Dhawan S., Sharma T., Batra D. (2019). Effect of Different Irrigating Solutions with Surfactants on the Microhardness and Smear Layer Removal of Root Canal Dentin: An in Vitro Study. J. Conserv. Dent. JCD.

[43] Sayin T.C., Serper A., Cehreli Z.C., Otlu H.G. (2007). The Effect of EDTA, EGTA, EDTAC, and Tetracycline-HCl with and without Subsequent NaOCl Treatment on the Microhardness of Root Canal Dentin. Oral Surg. Oral Med. Oral Pathol. Oral Radiol. Endod..

[44] Abdelrhman M., Mahraan A., Bayoumy A. (2023). Impact of Two Nano Irrigating Solutions on Microhardness of Root Canal Dentin. Egypt. Dent. J..

[45] Abdelgawad R., Fayyad D. (2017). Comparative Evaluation of Smear Layer Removal, Calcium Ions Loss and Dentin Microhardness after Different Final Irrigation Solutions. Egypt. Dent. J..

[46] Alyahya A.A., Rekab M.S., AL-Ostwani A.E.O., Abdo A., Kayed K. (2022). The Effect of a Novel Silver-Citrate Root Canal Irrigation Solution (BioAkt), Ethylenediamine Tetraacetic Acid (EDTA), and Citric Acid on the Microhardness of Root Canal Dentin: A Comparative In Vitro Study. Cureus.

[47] Taneja S., Kumari M., Anand S. (2014). Effect of QMix, Peracetic Acid and Ethylenediaminetetraacetic Acid on Calcium Loss and Microhardness of Root Dentine. J. Conserv. Dent. JCD.

[48] Souza M.A., Trentini B.M., Parizotto T.F., Vanin G.N., da Silva Piuco L., Ricci R., Bischoff K.F., Dias C.T., Pecho O.E., Bervian J. (2021). Influence of a Glycolic Acid-Based Final Irrigant for Photosensitizer Removal of Photodynamic Therapy on the Microhardness and Colour Change of the Dentin Structure. Photodiagn. Photodyn. Ther..

[49] Aranda-Garcia A.J., Kuga M.C., Chavéz-Andrade G.M., Kalatzis-Sousa N.G., Hungaro Duarte M.A., Faria G., Reis Só M.V., Faria N.B. (2013). Effect of Final Irrigation Protocols on Microhardness and Erosion of Root Canal Dentin. Microsc. Res. Tech..

[50] Nikhil V., Jaiswal S., Bansal P., Arora R., Raj S., Malhotra P. (2016). Effect of Phytic Acid, Ethylenediaminetetraacetic Acid, and Chitosan Solutions on Microhardness of the Human Radicular Dentin. J. Conserv. Dent. JCD.

[51] Ballal N.V., Mala K., Bhat K.S. (2010). Evaluation of the Effect of Maleic Acid and Ethylenediaminetetraacetic Acid on the Microhardness and Surface Roughness of Human Root Canal Dentin. J. Endod..

[52] Eldeniz A.U., Erdemir A., Belli S. (2005). Effect of EDTA and Citric Acid Solutions on the Microhardness and the Roughness of Human Root Canal Dentin. J. Endod..

[53] Saghiri M.A., Delvarani A., Mehrvarzfar P., Malganji G., Lotfi M., Dadresanfar B., Saghiri A.M., Dadvand S. (2009). A Study of the Relation between Erosion and Microhardness of Root Canal Dentin. Oral Surg. Oral Med. Oral Pathol. Oral Radiol. Endod..

[54] Chu C.-Y., Kuo T.-C., Chang S.-F., Shyu Y.-C., Lin C.-P. (2010). Comparison of the Microstructure of Crown and Root Dentin by a Scanning Electron Microscopic Study. J. Dent. Sci..

[55] Inoue T., Saito M., Yamamoto M., Nishimura F., Miyazaki T. (2013). Mineral Density of Coronal and Radicular Dentin. Dent. Med. Res..

[56] Sim T.P., Knowles J.C., Ng Y.L., Shelton J., Gulabivala K. (2001). Effect of Sodium Hypochlorite on Mechanical Properties of Dentine and Tooth Surface Strain. Int. Endod. J..

[57] Chuenarrom C., Benjakul P., Daosodsai P. (2009). Effect of Indentation Load and Time on Knoop and Vickers Microhardness Tests for Enamel and Dentin. Mater. Res..

[58] Thompson J.M., Agee K., Sidow S., McNally K., Lindsey K., Borke J., Elsalanty M., Tay F.R., Pashley D.H. (2012). Inhibition of Endogenous Dentin Matrix Metalloproteinases by Ethylenediaminetetraacetic Acid. J. Endod..

[59] Al-Omari W.M., Palamara J.E. (2013). The Effect of Nd:YAG and Er,Cr:YSGG Lasers on the Microhardness of Human Dentin. Lasers Med. Sci..

[60] Zaparolli D., Saquy P.C., Cruz-Filho A.M. (2012). Effect of Sodium Hypochlorite and EDTA Irrigation, Individually and in Alternation, on Dentin Microhardness at the Furcation Area of Mandibular Molars. Braz. Dent. J..

